# A case of paroxetine-induced galactorrhoea with normal serum prolactin level

**DOI:** 10.4103/0253-7613.70399

**Published:** 2010-10

**Authors:** Suddhendu Chakraborty, Debasish Sanyal, Ranjan Bhattacharyya, Subhendu Dutta

**Affiliations:** Department of Psychiatry, Calcutta National Medical College, Kolkata, India

**Keywords:** Paroxetine, selective serotonin reuptake inhibitors, galactorrhoea

## Abstract

The following case report highlights an interesting observation of paroxetine-induced galactorrhoea at therapeutic dosage and serum prolactin values of this patient comes out to be normal. This observation merits a systematic study to find the causal relationship of this unusual phenomenon and further explanation.

## Introduction

The selective serotonin reuptake inhibitors (SSRIs) are one of the most commonly used agents to treat depression, anxiety, obsessive compulsive disorders, etc. Among the SSRIs, paroxetine is preferred by the clinicians for its calming, sedating and comparatively lesser activating actions in the initial phase of treatment compared to other SSRIs like fluoxetine and sertraline.[1] The common side effects of paroxetine are gastrointestinal upset, sexual dysfunction, and prominent withdrawal reaction in the form of akathisia, dizziness, and restlessness upon sudden discontinuation. Galactorrhoea has been only rarely mentioned as a side effect of this drug. However, Egberts *et al*. (1997) reported an 8-fold higher risk of galactorrhoea upon usage of SSRIs, compared to other antidepressants.[2] Here we discuss a case of paroxetine-induced galactorrhoea with normal levels of serum prolactin.

## Case Report

A 32-year-old lady came to the psychiatry out-patient department with complaints of anxiety for the last 6 months. She felt extremely distressed and panicky in crowded places like railway station and market places to such an extent that she started avoiding going to these places. She also developed fear of heights and started avoiding going to high buildings and also a fear of empty rooms which led to significant distress in her social life. There were at least two incidents within a span of 1 month where the patient’s experiences met the criteria of a panic attack. The patient also complained of low mood, loss of interest in her daily household works, reduced sleep and appetite, and fatigability for the last 6 months. Her clinical examinations including the general survey and systemic examinations were all found to be normal. The routine investigations including blood count, sugar, urea, creatinine, liver function test, and thyroid profile were within normal limits. A provisional diagnosis of panic disorder with agoraphobia (with co morbid depression) was made in accordance to criteria led down in the Diagnostic and Statistical Manual of Mental Disorders with text revision (DSM IV-TR).[3]

She was prescribed 12.5 mg/day of paroxetine for the first 10 days, and then the dose was increased to 25 mg per day. The patient showed significant improvement in all the spheres on the follow up visit after 3 weeks. Her anxiety to crowded places and different social gatherings had subsided significantly, her sleep disturbance alleviated, and she had regained her confidence in participating in different social activities.

After 6 weeks of continuing with the therapy, she complained of milk secretion from both her nipples, the volume being significant. Clinical examination revealed galactorrhoea (i.e. non puerperal discharge of milk containing fluid from the breast). She was investigated for the common causes of galactorrhoea. The pregnancy test was negative. The clinical examination was inconclusive. She did not complain of any disturbance of vision or headache. There were no signs of raised intracranial tension. She had no history of any local surgery or herpes zoster infection. The thyroid profile was checked and was found to be normal. The serum FSH and DHEAS levels were normal. The serum prolactin level was 14.01 ng/ml (normal levels being 2.8 to 29.2 ng/ml). The magnetic resonant multiplanar imaging of brain (T1,T2 weighted and FLAIR sequences) did not show any significant abnormality.

Considering the above reports, paroxetine was assumed to be responsible for the galactorrhoea and was stopped following which the galactorrhoea had subsided completely within 7 days. Her prolactin levels were again found to be normal when assessed 7 days after stoppage of galactorrhoea. Repeat thyroid level estimation after 4 weeks showed normal levels.

## Discussion

Though galactorrhoea caused by the use of paroxetine has been reported earlier, the commonly perceived cause is hyperprolactinemia.[4] Hyperprolactinemia can be caused by two distinct mechanisms namely-presynaptic inhibition of dopamine discharge by serotonergic receptors or the direct stimulation of hypothalamic post synaptic serotonergic receptors.[2,5] The peculiarity of the case lies in the fact that serum prolactin levels were not raised. Only a very few such cases have been reported in the literature.[4,6–8] According to some researchers approximately 50% of the patients presenting with galactorrhoea may have normal serum prolactin levels.[9] The exact mechanism of galactorrhoea remains unknown in many cases. Hence more research is required to understand the true mechanism behind SSRI-induced galactorrhoea. Hence the clinicians should be aware that galactorrhoea can occur as a side effect of SSRI and may require stoppage of these otherwise necessary drugs in these patients.
